# Grand challenges in molecular cardiology

**DOI:** 10.3389/fmmed.2022.920039

**Published:** 2022-11-18

**Authors:** Leon J. De Windt

**Affiliations:** Department of Molecular Genetics, Faculty of Science and Engineering, Faculty of Health, Medicine, and Life Sciences, Maastricht University, Maastricht, Netherlands

**Keywords:** molecular cardiology, molecular therapy, vascular disease, valve disease, cardiac disease, arrhythmia

## Introduction

**FIGURE 1:**
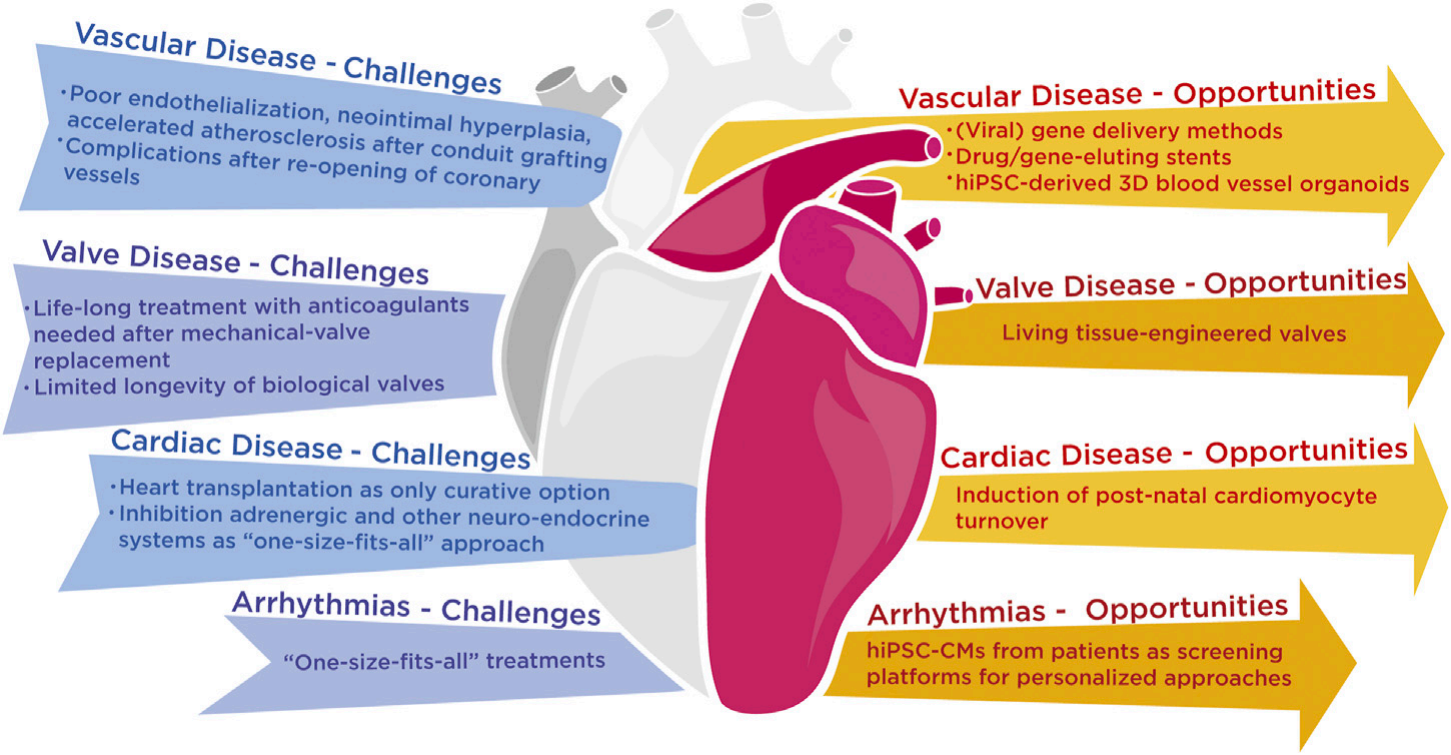
Grand challenges that the scientific and medical community are facing with the current state-of-the-art technology in vascular, cardiac and rhythm diseases and possible opportunities that lie ahead to resolve these challenges.

Despite technological breakthroughs, cardiovascular disorders remain the primary cause of death worldwide, with limited therapeutic options and a dismal prognosis (Ziaeian and Fonarow, 2016). Cardiovascular diseases roughly encompass all conditions that affect the vasculature including thrombotic or bleeding disorders, abnormalities of the valvular system, and all disorders that the heart as a pump or as an electrical organ can suffer from. For each cardiovascular disease type, future molecular challenges are briefly addressed below and visualized (Figure 1).

## Vascular disease

Blood arteries are essential for the normal homeostasis of our body’s tissues and organs, and vascular malfunction is linked to a variety of cardiovascular and neurological disorders. Flow-impaired lesions, impaired tissue oxygen supply, and clinical symptoms such as angina pectoris or intermittent claudication are all indications of atherosclerosis. Myocardial infarction (MI) and stroke are caused by unstable lesions that rupture and thrombosis, resulting in substantial morbidity and death.

In acute coronary syndromes and symptomatic chronic disease, a combination of technological advances such as coronary artery bypass grafting (CABG) and percutaneous coronary intervention (PCI), including stent insertion, and pharmacological interventions with dual antiplatelet therapy are now established therapies. However, conduit graft failure in CABG and restenosis or even thrombosis of stented arteries in PCI contribute to a high cumulative graft failure rate caused by poor endothelialization, neointimal hyperplasia, and accelerated atherosclerosis (Sharma et al., 2011; Lopes et al., 2012). New treatments are therefore still needed to inhibit the extracellular matrix and intimal hyperplasia, vascular smooth muscle cell proliferation and migration, and the release of substances from injured endothelial cells, inflammatory cells, and platelets, despite the discovery of drug-eluting stents (Sharma et al., 2011). The interaction of proteins and non-coding gene products that cause this pathogenic cascade of events, identified by experimental investigations or unbiased genome-wide association studies (Nikpay et al., 2015), will remain a focus of modern vascular biology.

Another unmet need is to address microvascular dysfunction and obstruction, which can occur even after the successful re-opening of an obstructed coronary vessel and likely underlies many diverse pathologies such as organ dysfunction in aging, hypertension, diabetes, dyslipidemia, insulin resistance, and chronic inflammatory diseases. Additional attention is warranted to further develop and implement various tools to measure microvascular function in addition to invasive technology as a direct measurement of coronary flow reserve using intracoronary Doppler or non-invasive approaches such as myocardial contrast echocardiography or cardiac magnetic resonance (Niccoli et al., 2016).

Translational interventions are focused on a range of (viral) gene delivery methods or drug/gene-eluting stents in a number of animal models, including complicated mouse models and big animal models like rabbits and pigs, where technical interventions are more straightforward (Robertson et al., 2012). Human induced pluripotent stem cells (hiPSCs) derived from patients with genetic vascular disorders can form blood vessel cells, and even 3D blood vessel organoids (Wimmer et al., 2019), which can help us to better understand how vascular cells interact with their surroundings and provide humanized disease models that can be used to develop new therapeutic strategies.

## Valve disease

Heart valves enable unidirectional blood flow during the cardiac cycle.

A transvalvular pressure gradient induces the passive opening and closing of the valves as the heart contracts and relaxes, resulting in alternating blood flow from the atria to the ventricles and from the ventricles to the great arteries. A valve is considered to be faulty when it fails to open properly (stenosis) or does not close completely (regurgitation). Valve disease is a healthcare problem of epidemic proportions due to an increasing burden of elderly patients with degenerative heart valve disease and a growing population of young adults with congenital heart disease involving complex valve anomalies. Although surgical valve replacement is the gold standard for advanced heart valve disease, none of the mechanical or biological heart valve replacements now available are ideal. Patients with mechanical valves must take anticoagulants for the remainder of their lives, and patients with biological valves must undergo reoperation owing to the limited longevity of biological valves (Shinoka et al., 1995).

Tissue engineering attempts to overcome these flaws by using live cells to produce a living valve replacement that can remodel and repair damage in response to functional demand. Engineers look at valve morphogenesis at the embryonic stage, when endocardial cushions are formed *via* the epithelial-mesenchyme transition, followed by cellular and matrix remodeling to produce adult cusps and leaflets. Living tissue-engineered heart valves could have the same functionality and durability characteristics as natural heart valves (Chester and Grande-Allen, 2020).

## Cardiac disease

Heart failure (HF) is a rising disease that results from a variety of reasons such as ischemic heart disease, hereditary cardiomyopathies, and hypertension, and is the leading cause of hospitalization in the world’s aging population. In clinical terms, HF is described as a condition in which the heart’s function is compromised, making it unable to satisfy the organism’s energy needs.

For individuals with severe HF, heart transplantation is the only true curative option. Contemporary HF treatment still relies on inhibiting the heightened activity of the adrenergic and associated neuroendocrine systems to unload the heart. Although no genuinely new therapies have been brought to clinical fruition since then, this 40-year-old idea has fueled the development of systemic medications. Because of a fundamental lack of understanding of HF disease mechanisms, HF therapy takes a generic, “one-size-fits-all” approach, ignoring distinct sub-types of acquired and hereditary forms of HF, as well as inter-individual differences caused by underlying genetic susceptibility, age, gender, and disease stages.

Heart muscle cells undergo maladaptive growth without an increase in cell number in response to persistent stress or damage, such as pressure- or volume overload, to temporarily sustain cardiac output, resulting in a detectable thickening of heart muscle walls. Cardiac remodeling is also accompanied by a slew of biochemical, molecular, metabolic, and extracellular alterations that cause pump performance to deteriorate over time rather than be preserved, culminating in overt heart failure and an increased risk of fatal arrhythmias. Cardiomyocytes, endothelial cells, fibroblasts, pacemaker cells, and inflammatory cells make up the heart’s diverse cell composition. The critical function of each cell type in the formation, homeostasis, and remodeling of the heart is becoming increasingly evident, as is the complicated interplay between cell types in the cardiac microenvironment (Frieler and Mortensen, 2015).

A deeper knowledge of the molecular basis of cardiac hypertrophy aids in clarifying the disease’s maladaptive characteristics and already provides novel therapeutic targets for future therapy to reduce the number of HF patients. Altered calcium handling, changes in metabolic patterns and gene expression, perturbation of individual cell fate and survival, and fibrosis-induced alterations in the extracellular environment characterize pathological hypertrophic remodeling. Increased neurohumoral activation (mostly endothelin 1, angiotensin II, and catecholamines) and an aberrant mechanical stretch cause a number of sensors (particularly G-proteins and strain-sensitive cellular components) to converge on a number of stress response pathways. Important contributors to cardiac remodeling are RNA-based signaling pathways and mediators, as well as epigenetic and other posttranslational modifiers. MicroRNAs and long noncoding RNAs, in particular, have been demonstrated to play an important role in proper cardiac development and as stress regulators in the adult heart. The first clinical trials on these therapeutic targets are now emerging (Taubel et al., 2021).

Although a variety of diseases can cause HF, the most common subtype is ischemic heart disease, which means that the majority of patients who acquire HF initially have (or had) an ischemic episode (e.g. a myocardial infarction). After an injury, the adult mammalian heart is unable to repair or replace destroyed cardiac tissue with new functioning muscle. Given the significant shortage of donor hearts for transplantation, new regenerative treatments to heal severely damaged hearts are urgently needed. Cell transplantation methods appear appealing, but attempts to accomplish this have had inconsistent outcomes at best (Sahara et al., 2015). A promising field is post-natal cardiomyocyte renewal/turnover in animals. According to recent studies from several laboratories, cardiomyocyte turnover continues throughout life in animals, including humans, prompting researchers to look at ways to boost human cardiomyocyte renewal (Eulalio et al., 2012).

## Arrhythmias

Our understanding of the genetic and molecular origins of both congenital and acquired cardiac arrhythmias has progressed dramatically during the last three decades. The processes that cause cardiac arrhythmias can be grouped by conduction abnormalities (ie, reentry) or increased or aberrant impulse production (i.e. focused activity). Reentry occurs around a fixed anatomic obstruction or in a substrate with functional characteristics that allow reentrant circuits to be initiated and maintained. Normal automaticity in the myocardium is not present. However, disease conditions can cause the resting membrane potential to depolarize to higher positive levels, resulting in aberrant automaticity such as early afterdepolarizations (EADs), which occur before full repolarization, and delayed afterdepolarizations (DADs), which occur after full repolarization. EADs are most characteristic of Purkinje-fiber tissue and ventricular tachyarrhythmias associated with HF and long QT syndrome, while DADs are most common during diastole and when cellular calcium levels are high. The identification of the first hereditary mutations in calcium regulation protein genes has provided the greatest evidence to date that abnormalities in intracellular calcium management can induce various heart arrhythmias (Landstrom et al., 2017). Patient-specific treatments to reduce excessively lengthy repolarization are now emerging using human iPSC-CMs from patients with hereditary LQTS, for example, as screening platforms to better understand arrhythmia-inducing mechanisms and test new therapeutic interventions (Limpitikul et al., 2017). 

A continuous attempt to adopt a growing range of high-throughput methods, such as next-generation sequencing techniques, to find novel biomarkers or susceptible targets at whole genome or even single-cell resolution is common to all cardiovascular disease domains. This is aided by a continuous lowering of the costs of reagents, equipment, and the concentration of knowledge in academia and the private sector assembled in platforms. The emergence of screening or sequencing platforms imposes increasing demands on statistical methods, bioinformatic tools, and computational expertise for the data analysis workflow and the management of the enormous amounts of data generated by these technologies. Artificial intelligence is already being used to make unbiased discoveries of cardiovascular medication targets and to decipher complicated data. To produce next-generation interdisciplinary specialists, the rapid deployment of computational tools will increasingly necessitate the re-education of cardiovascular scientists and the deliberate steering of younger generations of scientists toward computational skills. A molecular, disease-oriented strategy will be facilitated by existing and new resources such as clinical samples, patient-derived hiPSC lines or organoids, as well as centering multidisciplinary expertise in the molecular cardiovascular field, with the ultimate goal of implementing precision medicine sooner rather than later.
